# Multimodal Hybrid Deep Learning Approach to Detect Tomato Leaf Disease Using Attention Based Dilated Convolution Feature Extractor with Logistic Regression Classification

**DOI:** 10.3390/s22166079

**Published:** 2022-08-14

**Authors:** Md Shofiqul Islam, Sunjida Sultana, Fahmid Al Farid, Md Nahidul Islam, Mamunur Rashid, Bifta Sama Bari, Noramiza Hashim, Mohd Nizam Husen

**Affiliations:** 1 Faculty of Computing, Universiti Malaysia Pahang, Kuantan 26300, Pahang, Malaysia; 2 Department of Computer Science and Engineeering, Islamic University, Kushtia 7003, Bangladesh; 3 Faculty of Computing and Informatics, Multimedia University, Cyberjaya 63100, Selangor, Malaysia; 4 Faculty of Electrical and Electronics Engineering Technology, Universiti Malaysia Pahang, Pekan 26600, Pahang, Malaysia; 5 Malaysian Institute of Information Technology, Universiti Kuala Lumpur, Kuala Lumpur 50250, Selangor, Malaysia

**Keywords:** dilated CNN, filtering, logistic regression, segmentation, tomato leaf disease, feature extraction

## Abstract

Automatic leaf disease detection techniques are effective for reducing the time-consuming effort of monitoring large crop farms and early identification of disease symptoms of plant leaves. Although crop tomatoes are seen to be susceptible to a variety of diseases that can reduce the production of the crop. In recent years, advanced deep learning methods show successful applications for plant disease detection based on observed symptoms on leaves. However, these methods have some limitations. This study proposed a high-performance tomato leaf disease detection approach, namely attention-based dilated CNN logistic regression (ADCLR). Firstly, we develop a new feature extraction method using attention-based dilated CNN to extract most relevant features in a faster time. In our preprocessing, we use Bilateral filtering to handle larger features to make the image smoother and the Ostu image segmentation process to remove noise in a fast and simple way. In this proposed method, we preprocess the image with bilateral filtering and Otsu segmentation. Then, we use the Conditional Generative Adversarial Network (CGAN) model to generate a synthetic image from the image which is preprocessed in the previous stage. The synthetic image is generated to handle imbalance and noisy or wrongly labeled data to obtain good prediction results. Then, the extracted features are normalized to lower the dimensionality. Finally, extracted features from preprocessed data are combined and then classified using fast and simple logistic regression (LR) classifier. The experimental outcomes show the state-of-the-art performance on the Plant Village database of tomato leaf disease by achieving 100%, 100%, 96.6% training, testing, and validation accuracy, respectively, for multiclass. From the experimental analysis, it is clearly demonstrated that the proposed multimodal approach can be utilized to detect tomato leaf disease precisely, simply and quickly. We have a potential plan to improve the model to make it cloud-based automated leaf disease classification for different plants.

## 1. Introduction

The detection of plant diseases is the foundation for crop disease prevention and crop quality assurance. Traditional plant disease detection systems rely heavily on human observation, resulting in low detection efficiency, generability and reliability. Farmers with a lack of technical competence and agricultural professionals are unable to serve the field at all times, causing them to overlook the most effective preventative opportunities. In recent times, image processing [1], pattern recognition [2,3], computer vision [4,5] and text [6,7,8,9] or video [6,10] analysis have fast advanced outcomes in recent years. A machine learning-based intelligent disease detection approach provides a means for effectively resolving agricultural concerns [11,12].

Tomato is considered the most significant and widely consumed crop after potato [13]. The farmland area used for horticultural crops has expanded by 164% in the last four decades. Recently, worldwide tomato production reached over 180 million tonnes [14,15]. China by far is the world’s top tomato grower, accounting for 31% of worldwide tomato yield. However, every year a vast amount of tomato plants are affected by numerous leaf diseases. This results in lower crop production, which affects human health and livelihood, as well as financial stability [16]. Therefore, it is very crucial to detect tomato leaf disease accurately in the early stage to decrease crop losses and assure optimal growth. Plant leaf diseases are traditionally classified by trained experts by doing a visual investigation of plant leaf tissues. These conventional techniques are very time-consuming, expensive, and expertise-dependent [17]. In recent years, machine-learning-based recognition techniques for crop disease identification became popular for their successful application [18]. Moreover, in the field of computer imaging, the conventional deep learning (DL) algorithms are the most widely used approach for the automated identification of plant diseases [17]. Neural Networks [19], K-nearest Neighbors (k-NN) [20], Niave Bayes [21], Logistic Regression [22], Random Forest (RF) [23], Support Vector Machines (SVM) [24], as well as adaptive boosting [20] are perhaps the most prominent conventional computer-based techniques used for plant disease categorization. Traditional machine learning approaches rely significantly on the features individuals provided. These traits are painstakingly extracted by the expert, making these procedures costly, as well as time-consuming. On the other hand, the limitations of the handcrafted features technique can be readily overcome with the automatic extraction of features utilizing deep learning (DL). Therefore, the DL methods are widely employed for plant disease categorization [25] because of its high performance. Among the wide range of DL methods, a deep convolutional neural network (CNN) is most widely used for crop disease detection [26]. An improved YOLOv5 algorithm of deep learning has been used in the detection of plant diseases [27]. However, the deep learning methods require a large amount of training data and a considerable amount of time for training. On the other hand, the performance of standard machine learning can be outperformed compared to deep learning whilst providing a limited amount of training data. Moreover, in terms of computing resources, deep learning is more expensive than traditional machine learning techniques. Actually, the machine learning algorithm is able to perform faster classification and deep learning performs well in feature extraction and classification.

For these considerations, this study has combined the DL with the ML method. In the initial stage, all image are preprocessed (Filtering and Segmentation) to generate the synthetic image using CGAN. At first, we used the attention-based dilated CNN to extract the feature from the tomato leaf disease images. During the feature extraction process, the hidden layer in deep learning models allows them to learn hierarchical representations. Deep architectures can select discriminating representations from model training that aid with exact predictions based on the training data in later classification phases. Finally, the ML method LR (Logistic Regression) is utilized to classify the extracted feature. The proposed tomato leaf disease detection approach is divided into three phases: fixed feature extractor using attention-based dilated CNN as fusion features, dimensionality reduction using normalization, and training of a logistic regression classifier. Furthermore, we also developed Logistic Regression (LR), CNN with LR (CNN-LR), and the proposed attention-based dilated CNN with LR (ADCLR) model to test the robustness and applicability of the proposed tomato leaf detection approach. The main contribution of this study is as follows:

1. In this study, we have introduced sequential image pre-processing steps. The tomato leaf images have been pre-processed using the color conversion, filtering method for denoising the images. To handle the larger features, we have used the bilateral filter method which helps to make images smoother with fine spatial parameters. Furthermore, the noises have been removed from the filtered data using the fast and simple Otsu segmentation method. Then, we use the CGAN model to generate synthetic image from the image to handle imbalance and noisy or wrongly labeled data to obtain good prediction results.
2. To extract the most informative feature in a short time, we have designed a lightweight dilated CNN architecture and attention mechanism in which the multiple hidden layers of the architecture allow them to learn hierarchical representations from the images. Then, the extracted features have been classified using the fast and simple logistic regression model.
3. To check the validation and robustness of the proposed hybrid architecture, we have also implemented eleven popular transfer learning algorithms on the same dataset and compared the performance with the proposed ADCLR model. The experimental analysis clearly shows that the proposed hybrid ADCLR provides superior performance for detecting tomato leaf disease.

**Figure 1:**
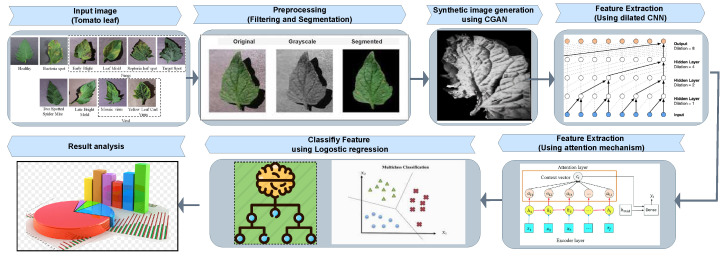
The overall framework of the proposed approach.

The basic flowchart of our proposed approach is shown in Figure 1. At first, the input images are preprocessed using filtering and segmentation methods. Then, we use the CGAN model to generate synthetic image. Synthetic image is generated to handle imbalance and noisy or wrongly label data to obtain good prediction results. Then, the synthetic images are used in the attention-based dilated CNN layer to extract the advanced features. Finally, the logistic regression model is used to learn the extracted features and classify the tomato leaf disease images accurately.

The remainder of the paper is laid out as follows: Section 1 conducts a state-of-the-art survey and explains the study’s objective. The data and dataset preprocessing are introduced in Section 2. The experimental setup, technique, and development of all section of the proposed method are described in Section 3. The results are presented in Section 4, followed by an informative discussion, before the conclusion is presented in Section 5.

## 2. Related Study

Nowadays, intelligent approaches for plant leaf disease detection have shown great successful applications in early diagnosis. The researchers have developed several strategies for automatic plant disease classification. In recent years, machine learning or deep learning approaches are widely adopted methods for the early diagnosis of plant disease.

### 2.1. Machine Learning Methods

To obtain a high-performing model, the researchers have used several data preprocessing steps such as color conversion, edge-based segmentation, filtering and segmentation. Furthermore, image analysis, shape, size, augmentation, and color conversion are used to extract the feature from the segmented images. Then, traditional machine learning methods have been utilized to detect plant diseases efficiently [28].

For example, Hlaing et al. [29] developed a feature extraction approach based on the Johnson SB distribution and a scale-invariant feature transform (SIFT). The proposed approach was used to extract SIFT and color statistic features, which were then fed into a multi-class support vector machine classifier for categorization. For tomato disease categorization, the proposed approach got an accuracy score of 85.1%. In [30], a novel method based on the concatenation of various features was presented. The Hue moments, Haralick, and color histograms were extracted and then combined. For tomato leaf disease categorization, the retrieved feature was input into the decision tree as well as RF classifiers. They achieved a maximum of 94% classification accuracy with the random forest method. Kalyoncu et al. [31] proposed a unique plant leaf disease categorization approach based on numerous characteristics. A digital image of a leaf is used to extract the shape, textural and geometric, as well as color properties. In particular, sorted uniform LBP is presented as a novel local binary pattern (LBP) alternative for describing leaf texture. A machine learning method using the SVM algorithm got very low accuracy of 85.02% but this method is fast [29]. The discriminant classifier (LD) is being used to classify the data once it has been combined with the retrieved characteristics. This approach was tested on three different datasets: ICL, Flavia, and Swedish. The average accuracy of ICL, Flavia, and Swedish was 86.8%, 98.6%, and 99.5%, respectively. The authors of [32] proposed a semi-automated technique for soybean disease detection based on color and texture characteristics. In their approach, they used a total of 4775 images for classification with the SVM method (90% accuracy).

### 2.2. Deep Learning Methods

In the past couple of years, researchers have widely focused on deep learning methods due to their successful application for plant disease diagnosis. In this sub-section, tomato leaf disease detection-related studies based on the deep learning method have been highlighted. Batool et al. [33] presented a tomato leaf disease identification method. In their study, the AlexNet pre-trained model was used for feature extraction. Then, the kNN method was used to classify the extracted feature. This achieved a maximum of 76.1% testing accuracy. Another study [34] developed a transfer learning model (MobileNetV2) for tomato leaf disease classification. To improve the model performance, they utilized the fine-tuned strategy of the MobileNetV2 model, and achieved impressive performance (90% accuracy) with the fine-tuned model. Agarwal et al. [35] proposed a CNN approach for tomato disease diagnosis (91.2% accuracy). In [36], an inception method combined with dilated convolution was used to identify 26 diseases of 14 different crops. They achieved a maximum of 99.37% classification accuracy with the PlantVillage dataset. A novel CNN model with eight hidden layers was introduced in [37] for tomato plant disease detection. They achieved 98.4% classification accuracy with the PlantVillage dataset. The authors [38] proposed a nine-layer CNN model to detect 39 types of the plant leaf. To enhance the performance of their model, they used different data augmentation techniques and finally achieved 96.46% accuracy on the PlantVillage dataset of tomato leaf disease. Nithish et al. [39] developed a pre-trained deep learning method (ResNet-50). The ResNet-50 architecture was fine-tuned to successfully categorize the six classes of leaf disease and achieved a 97% average classification accuracy.

### 2.3. Deep Learning with Machine Learning

For the particular task classification to be more effective, the researchers have developed a hybrid model where they combined the ML-ML, ML-DL, or DL-DL methods. Due to the excellent feature learning capabilities of the DL methods, some recent studies used the DL layers to extract the feature from the data [40]. Several hidden layers of the DL model have the capabilities to select the discrimination feature more effectively. Fisher et al. [41] proposed an approach where the image’s features were extracted using the CNN network. The images were classified using the RPN and Fast-RCNN by constructed feature maps. Furthermore, VGG networks are commonly used with faster RCNN. ResNet [42] has more convolution layers than VGG16 and can use convolution to extract more object information. ResNet has a layer-skipping structure that allows it to skip one or more layers immediately. It addresses the issue of gradient disappearance caused by layer stacking. On the other hand, the VGG16 network is unable to extract detailed aspects of tomato leaf diseases [43]. A feed-forward neural network with such a residual connection is used to create the deep residual network. The identical mapping function of the skipping structure allows the output with one stage to be used as the source of a subsequent layer. The benefit of this method is that no other variables are imported, as well as the calculation time not being greatly increased. Gradient disappearance [44] induced by expanding the number of neurons in the hidden layer is prevented by employing cross-layer operations and reusing intermediary features. As a result, the deep residual network is crucial in the field of recognition. In addition, deep residual networks are frequently used in defect detection as well as fault-tolerant control [45,46]. Some studies have shown that the ML with DL approach provides slightly higher performance than conventional ML or DL method. For example, the MobileNet and NasNet feature extractor with Logistic Regression achieved 97% accuracy [28]. On the other hand, recently, the dilated CNN mechanism has become more popular because of its effective and fast feature learning capabilities [47].

Another attention-based method proposed by Devi et al. [48] that used the Salp Swarm Algorithm to classify tomato leaf disease. This method got 97.56% accuracy to predict five types of tomato leaf disease from plan village data. The limitation of this method is that its performance is not high and it faces some computational complexity. On the other hand, a method that utilized the Lightweight Attention-Based CNN mechanism [49] to classify ten types of tomato leaf disease has 99.34% accuracy but it has slightly higher time complexity than conventional methods such as CNN [37] and SVM [29]. In 2022, Zhao et al. [50] developed a method utilizing spatial attention with CNN for real time leaf disease detection. This method has 95.20% accuracy but this method needs to be adaptable by increasing its generability.

## 3. Materials and Methods

### 3.1. Data Description

**Figure 2:**
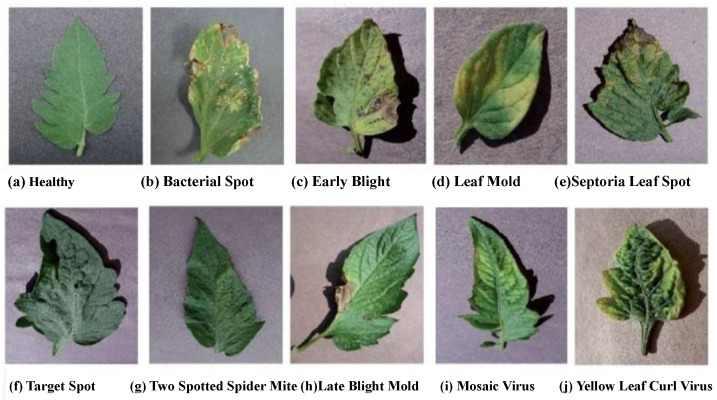
The sample images of the tomato leaf disease.

In this experimental analysis, a well-known PlantVillage dataset crosscheck the citation [51] was used to detect the tomato leaf disease. Hughes and Salathe et al. [52] generated the PlantVillage collection, which includes 54,309 label images for 14 different species and 38 different types of healthy and leaf disease images. From the entire dataset, we used 15,989 images of tomato leaves grouped into ten classes. Then, the selected images were resized into 256 × 256 pixels. The resized images were then normalized. The normalized procedure helps to speed up the training procedure by reducing the computational complexity. To test the validation of the proposed architecture, we used a total of 1000 new images during testing (100 images for each class) from 54,309. The detailed data description is shown in Table 1. Figure 2 shows the basic image samples of tomato leaf disease.

### 3.2. Data Preprocessing

In the preprocessing phase, we added a label for each image depending on the prefix of their filename. Overall preprocessing task is presented sequentially in the Algorithm 1. At first, the images were segmented using Bilateral filtering and Ostu’s image segmentation process. Before the segmentation process, two-color converting functions were applied where the sequence of the image’s color-space was lost and only the brightness and saturation for each pixel were kept. After that, data normalization was accomplished by calculating the mean difference between each pixel and dividing the result by the standard deviation. The images were normalized to make it easier for the error function, which is typically not convex, to identify the global minimum. Decreasing the range of inputs for training variables also aids the backpropagation algorithm’s efficiency. Python’s random shuffle technique, which is a command algorithm based on an arbitrary number generator, was used to shuffle the data. The order of the images was originally sequential after the application, but it is now mixed throughout the collection.

A multilevel categorization is a strategy that uses more than two labels. Every label in this classification is not exclusive. For each sample, the classification method yields only one degree. In actuality, multi label classification of leaf disease is used to label categories of tomato leaf disease in one or more types. We focused on the image’s multilevel classification of tomato leaf disease. We conducted a multilevel study on the D data set. For further information on data collection, see Area F of this technical section. The data includes visual features of tomato leaf disease and each declaration set on the vector stage. The labeling of the tomato disease image is presented in Equation (1).

(1) $${D=\left(E,F\right)|F\in Image,E\in \left(0,1\right)}^{L}$$

Here, *F* is the Tomato leaf disease images, dataset *E* with *L* (Number of Tomato leaf disease class label) target disease class, and the Tomato leaf disease categories indicated are increased level *L* which is ten.
**Algorithm 1** Preprocessing Algorithm for ADCLR Model1:*Input: Dataset of tomato leaf disease*2:*Result:Preprocessing of tomato leaf disease image data, with number of image N*3:**for** Each iteration i in range(0,N) **do**4:   *Resize to dimension (256 × 256)*5:   *Pre-processing to resize the image (224 × 224)*6:   *Normalize pixel values [0, 1]*7:   *Standardize pixel values to (256 × 256)*8:**end for**9:**if** Image set size is (224 × 224) **then**10:   *Normalize pixel value [0, 1]*11:   *Standardize pixel values to (256 × 256)*12:**end if**13:**if** Image set size is (224 × 224) **then**14:   *Perform Image Filterng with Bilateral Filter*15:   *Complete Otsu segmentation*16:**end if**17:*Generation of Preprocessed image of tomato leaf*

### 3.3. Image Filtering

The bilateral image filtering method is used in our method. In this filtering, any input image (a) is converted to a smoothed form by the bilateral filter (b). Then, most texture, noise, and small details are removed, but broad sharp edges are preserved without being blurred. A bilateral filter is a non-linear image smoothing filter that preserves edges while lowering noise [53]. It uses a weighted mean of intensity data from surrounding pixels to adjust the brightness of each pixel of the disease image. A Gaussian distribution could be used to calculate this weight. The weights are determined not just by the Euclidean distance between pixels, but also by the radiometric variations (color intensity, depth distance, etc.). Sharp edges are preserved as a result. The computation of Bilateral image filtering is utilized with the Equation (2).

The bilateral filter is defined as

(2) $$F^{Filter}\left(x\right)=\frac{1}{W_{p}}[\sum_{Cocf_{i}\in \Omega }F\left(cocf_{i}\right)f_{r}((||F\left(cocf_{i}\right)-F\left(cocf\right)\left||)\right)g_{s}\left(|\right|\left(cocf_{i}-cocf\right)\left||)\right]$$

and normalization term, $W_{p}$ is defined as the Equation (3).

(3) $$W_{p}=\sum_{Cocf_{i}\in \Omega }f_{r}(||I\left(cocf_{i}\right)-F\left(cocf\right)\left||\right)g_{s}(||cocf_{i}-cocf||)$$

Here, $F^{Filter}$ indicates a filtered image of the tomato leaf disease data, *I* is considered as the original image to be filtered, cocf indicates the current pixel coordinate to filter, Ω presents a window centered in the $cocf_{i}$ so $cocf_{i}\in \Omega$ is another pixel. $f_{r}$ indicates the smoothing intensity difference. When smoothing disparities in positions, $g_{s}$ is the spatiotemporal (or domain) kernel (this functional method can be a Gaussian distribution).

In the filtering of the image of tomato leaf disease, the spatial closeness (using spatially kernel $g_{s}$) and the intensity difference (using the range kernel $f_{r}$) are used to give the weight $W_{p}$. Imagine a pixel at (i,j) that is needed to be denoised inside an image by using its neighbors, and one of its neighbors is at (i,j) (k,l). Each assigned pixel (k,l) to remove the noise the pixel (i,j) is provided by the following Equations (4) and (5), presuming the range, as well as spatial kernels, are Gaussian kernels.

(4) $$\omega \left(i,j,l\right)=exp\left(-\frac{{\left(i-k\right)}^{2}+{\left(j-l\right)}^{2}}{2\sigma_{d}^{2}}-\frac{F\left(i,j\right)+F{\left(k,l\right)}^{2}}{2\sigma_{r}^{2}}\right)$$

Here, $\sigma_{d}$ and $\sigma_{r}$ both indicate the smoothing parameters, and F(i,j) and F(k,l) presents intensity of pixels (i,j) and (k,l).

(5) $$F_{D}\left(i,j\right)=\frac{\sum_{k,l}F\left(k,l\right)w\left(i,j,k,l\right)}{\sum_{k,l}w\left(i,j,k,l\right)}$$

Here, $F_{D}$ presents the denoised pixel intensity of the pixel (i,j). The bilateral filter steadily approaches like Gaussian convolution relatively closely as the ranged parameter $\sigma_{r}$ grows. Actually, the range of Gaussian expands and compresses, which implies that it becomes nearly constant through the image’s intensity intervals. The larger features become smoother as the spatial parameter value of $\sigma_{d}$ is increased.

### 3.4. Image Segmentation

Object and boundary (lines, curves, etc.) inside images are often identified via the image segmentation method. Image segmentation is typically used to assign a label to each image pixel because pixels with nearly identical labels have similar characteristics. Image segments are used to reduce the complexities of an image, making subsequent processing and analysis easier. In layman’s terms, segmentation is the process of labeling pixels.

For this purpose, we have used the Otsu segmentation approach to segment the tomato leaf disease images. Automatic image thresholding is performed using Otsu’s [1]. This method generates a single intensity threshold that divides pixels into two different classes: foreground as well as background. This limit is set by reducing intraclass intensity variation. The technique looks for a threshold that minimizes intraclass variance, which is defined as the weighted combination of the classes’ variances as given in Equation (6).

(6) $$\sigma_{P}^{2}\left(t\right)=P_{0}\left(t\right)\sigma_{0}^{2}\left(t\right)+\;P_{1}\left(t\right)\sigma_{1}^{2}\left(t\right)$$

In the above Equation (6) $P_{0}$ and $P_{1}$ indicates the class probability with threshold value *t* difference and $\sigma_{0}^{2}$ and $\sigma_{1}^{2}$ presents variance.

Here, $P_{0},1\left(t\right)$ is measured by the histogram bins *L*, o(i) indicates previous probability as computed in Equations (7) and (8):

(7) $$P_{0}\left(t\right)=\sum_{i=0}^{t-1}o\left(i\right)$$

(8) $$P_{1}\left(t\right)=\sum_{i=t}^{L-1}o\left(i\right)$$

The minimizing and maximizing of the intra-class variance is equivalent as computed in Equation (9).

(9) $$\sigma_{b}^{2}\left(t\right)=\sigma^{2}-\sigma_{P}^{2}\left(t\right)=P_{0}\left(t\right){\left(\mu_{0}-\mu_{T}\right)}^{2}+P_{1}\left(t\right){\left(\mu_{1}-\mu_{T}\right)}^{2}=P_{0}\left(t\right)P_{1}\left(t\right){\left[\mu_{0}\left(t\right)-\mu_{1}\left(t\right)\right]}^{2}$$

Generally, Otsu’s technique initializes the value of $P_{i}$ and μ, then updates their levels based on threshold levels to obtain the intended $\sigma_{b}^{2}\left(t\right)$ threshold. The Otsu threshold is indeed a fast as well as a simple algorithm that works with histograms (that are 256-element integer or float vectors).

**Figure 3:**
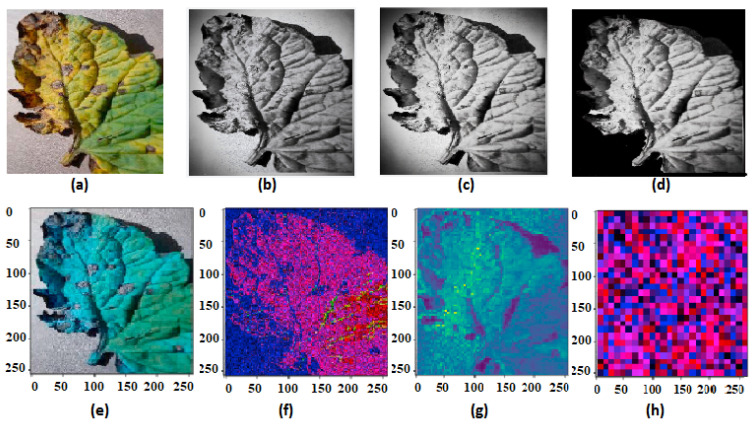
(**a**) Original image; (**b**) Gray scale image; (**c**) BF filtered image; (**d**) Otsu’s image segmented image; (**e**) extracted features at first dilated convolution layer; (**f**) extracted features at the second hidden layer of dilated convolution; (**g**) extracted feature at the third hidden layer of dilated convolution; and (**h**) extracted features at attention layer.

Figure 3 clearly shows the preprocessed result image and feature extraction result using our method.

### 3.5. Synthetic Image Generation

This method uses the Conditional Generative Adversarial Network (CGAN) model to generate synthetic images from preprocessed images. To deal with imbalance and noisy or worn-out label data, a synthetic image is generated. This aids in training the model because it can predict well.

Initially, the Generative Adversarial Network (GAN) was developed in 2014 [54]. The GAN is built on the concept of having two neural networks fight in a zero-sum mechanism. Therefore, it is adversarial, in which the loss with one network benefits of another directly, as well as vice versa. To use picture generation for instance, there are distinct networks in this work. A generating network that makes images as well as a discriminator network that classifies the inputs as true or bogus. A gradient descent technique is used to update the gradients of each network after each train batch, as is the case with most deep learning approaches. Because the generator network’s output goes directly further into discriminator network, the combined networks’ training is automated through competition. A score can be determined using categorical cross-entropy as follows:

(10) $$Loss=E_{x}\times \left[log\left(Dis\left(x\right)\right)\right]+E_{z}[log\left(1-Dis\left(Gen\left(z\right)\right)\right]$$

(11) $$Extended_{Loss}=E_{x}\times \left[log\left(Dis\left(x/y\right)\right)\right]+E_{z}[log\left(1-Dis\left(Gen\left(z/y\right)\right)\right]$$

In the equation Equation (10), where ($E_{x}\times \left[log\left(Dis\left(x\right)\right)\right]$) indicates the recognition of real images and the ($E_{z}[log(1-Dis\left(Gen\left(z\right)\right)$) is used for the recognition of fake tomato leaf disease images. An additive noise input to a generator that starts as a real input from the dataset. The term Dis(x) is used to detect false photos $E_{x}$ or $E_{z}$ since it calculates the probability that a given piece of data is real. Because the discriminator’s source is Generator G’s result while confronted with a random source vector, *z*, Dis(x) is replaced by Gen(z) in the remaining half of the equation. Because the generator’s goal is to maximize or minimize the loss function with the Equation number (10), whereas the discriminator’s goal is to minimize it, this is regarded as the function to minimax loss.

**Figure 4:**
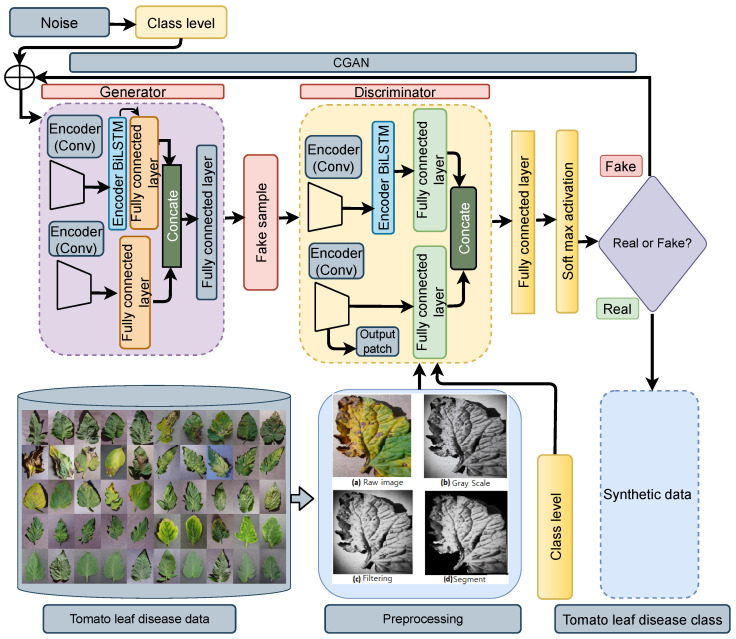
Synthetic Image Generation using GAN.

A Conditional Generative Adversarial Network (CGAN) [54] is an extended version of the previous GAN model that works on given number of disease class. This mechanism of image augmentation is also used in fruit classification [55]. The generator now aims to learn to generate images belonging to one of ten classes of tomato leaf disease. Equation (10) is expanded as the Equation number (11), data objects and its label is ensured. As a result, Gen(z/y) is the result of the generator having random vector given class *y* labeling, and Dis(x/y) is the discriminator’s confidence that *x* is real provided class label *y*. The production of objects corresponding to several classes is enabled by the minute difference in topological from a GAN, as shown in Figure 4. When the dataset was fed into a conditional GAN, the system would learn to generate fake tomato leaf disease images by training on actual tomato leaf disease photos, requiring two networks to produce either class. The networks in question would also need to train independently.

### 3.6. Proposed Hybrid Classification Model

**Figure 5:**
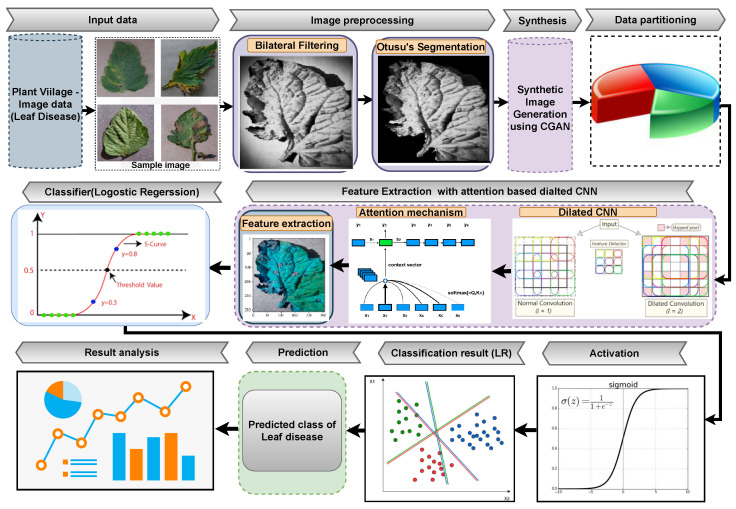
The overall procedure of the DCLR model.

In this study, we have designed a hybrid deep learning model (ADCLR) to detect tomato leaf disease more efficiently. A new hierarchical attention network with a dilated convolutional neural network (CNN) is used with multiclass in our tomato leaf disease image categorization technique. At first, we take the publicly available dataset, followed by preprocessing of all data and then synthetic image generation. The outcome of the preprocessing is then sent to a new vector routing algorithm for extracting feature information from the deep layer of the dilated CNN and then to the attention layer. Finally, the Logistic Regression Classifier is used to classify the extracted features. Our proposed model used categorical cross-entropy with the Adam optimizer and ROC assessment approach. A multilevel disease identification module incorporates these layers. The overall sequential process flow chart of our model is shown in Figure 5. The main Algorithm 2 of our ADCLR method describes the overall process clearly.
**Algorithm 2** General Algorithm of our ADCLR Model1:*Input: Dataset of tomato leaf disease* STATE *Output: Prediction of tomato leaf disease image*2:*Initialization: N:= Number of image, C:= Class label*3:**for** Each iteration i in range(0,N) **do**4:   *Take each image i*5:   *Pre-processing to resize the image i as the resolution of (224 × 224)*6:   *Normalize pixel values [0, 1] of the image i*7:   *Standardize pixel values to (256 × 256) of the image i*8:   *Image filtering with bilateral filtering method of the image i*9:   *Segmentation of image with Otsu method of the image i*10:     *Generation of preprocessed image*11:**end for**12:**for** Each iteration j in range(0,N) **do**13:  *Take preprocessed image j*14:  *CGAN based Generation of synthetic image j of tomato leaf with disease label C*15:**end for**16:*Initialization of parameter*17:*DC_k_*:= Extracted feature by Dilated *CNN*18:$A_{k}:=\;Extracted\;relevant\;feature\;by\;Attention\;mechanism$19:**for** Each iteration k in range(0,N) **do**20:  $Take\;synthetic\;image\;S_{k}\;and\;its\;class\;label\;C$21:  $Use\;dilated\;CNN\;to\;extract\;features\;DC_{k}\;of\;the\;image\;s_{k}$22:  *Use attention mechanism to capture relevant feature* $A_{k}$*from dilated CNN feature*$DC_{k}$23:  *Use logistic regression classifier to classify image with features* $A_{k}$*to the target classC*24:  *Generate prediction score of each image for target class C*25:**end for**26:*Evaluate all the prediction of tomato leaf disease image*

The following are the technical strengths of our proposed study:

Our method has the following steps:

1. Initially, the inputted data is preprocessed by color conversion, filtering, and denoising. Bilateral filtering is used and can handle larger features to make the image smoother with fine spatial parameters. Noise from the preprocessed filter is also removed by the fast and simple Otsu segmentation method.
2. Then, we use the Conditional Generative Adversarial Network (CGAN) model to generate synthetic image from the image those are preprocessed in previous stage. The synthetic image is generated to handle imbalance and noisy or wrongly labeled data to obtain good prediction results.
3. Then, the synthetic image is sent to our proposed ADCLR model. In the ADCLR model, the attention-based Dilated CNN is used to extract the informative feature extraction. Dilated convolution has the advantage of capturing the level of internal sequence data first by increasing the region of the convolution kernel without raising the model’s parameter amount. The attention layer simply concentrates on the memory block, instead of focusing on the entire feature space, attention mechanism has the benefit of dramatically reducing the number of parameters and sharing the weights among diverse regional places.
4. After that, the ADCLR method is trained with the training dataset and it tests the model robustness with the validation dataset. The Logistic Regression classifier is used to classify the images based on the extracted feature. Logistic regression classifier is simple, takes less time in training, and it performs well in multiclass prediction.
5. Finally, the validation of the proposed model is tested with different performance evaluation metrics and comparison on disease image.
6. To test the validation and effectiveness of the proposed approach, we also implemented eleven popular deep learning methods with the dataset, whereas our proposed method shows superior performance.

#### 3.6.1. Dilated CNN Layer for Feature Extraction

**Figure 6:**
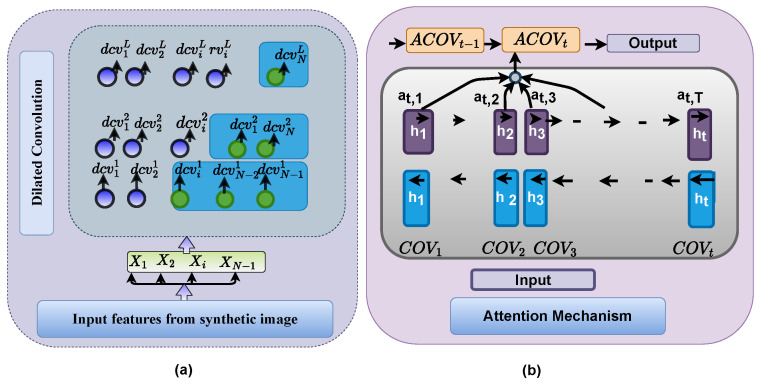
(**a**) Dilated CNN and (**b**) Attention Mechanism.

In this phase, a few feature variables such as color features are regarded because their visual color difference recognizes whether the plant leaf is exposed to the virus by the disease or not after a human perception in the precise system. In the Dilated CNN network, the multiple hidden layers allow the model to learn the discriminatory feature more efficiently. Deep learning, unlike machine learning, learns leaves with diseases and classifiers automatically, resulting in the machine learning’s efficiency in such contexts. The methodology maintains a strategy from the demand for humans to ideas by accumulating information from experience, empowering the computer to understand complicated ideas by creating them out of smaller complexes. The outputs of the multiple layer levels of a dilated convolutional neural network and attention layer are responsible for feature extraction and selection, as shown in Figure 6.

In the ADCLR model, this stage is one of the most crucial stages. The dilated convolution layer’s deep depth tries to find hierarchical, granular quality features that can be used to describe compositional feature information. Our feature findings are pooled and delivered to a Dilated CNN layer to produce DCV output, unlike typical CNNs, which perform dilated convolution operations instantly. For the few blocks of convolution given in Figure 6. Operational steps of the dilated CNN is given in the feature extraction Algorithm 3. In Figure 6a, each green colored dot indicates that this block is the block where selected convolution is performed. We define it as follows. As a consequence, the deep CNN layer generates the following set of variables as the Equation (12):

(12) $$dcv_{i}=dconv_{1},dconv_{2},\ldots ,dconv_{n}\in R^{n\times d^{∼}}$$

here, dcv is the output of the dilated convolution and *d* indicates the input sequence as (13).

(13) $$DCV^{1}=\left[dcv_{1}^{l},dcv_{2}^{l},\ldots ,rv_{n}^{l}\right]\in R^{n*d^{∼}},l\in \left(1,L\right)$$

In this Equation (13), *L* is the overall convolutional box, and the blocks filter has a degree of *k*. Let us focus on block *l*-th number alone.

(14) $$W^{l}\in R^{k*w*k},W^{l}\in R^{k*w*d^{∼}}$$

This filtering matrix W employs that the operation held in *k* time, as well as weight w vector. The two adjacent blocks can be changed as seen in Equation (15) below.

(15) $$DCV=F(W^{1},DCV^{l-1})$$

It is just a sliding of filtering with a window used to *w*-length input, where *f* represents linear algebra expression. Normally, $dcv_{1}^{l}\in DCV^{l}$ is computed as the Equation (18).

(16) $$dcv_{t}^{l}=ReLU(W^{l}⊕{\left[dcv_{t+1r}^{l-1}\right]}_{t=0}^{w-1})$$

Here, the ⊕ sign indicates convolution, *r* presents the level of the deep layer in the dilation. The ReLU with all blocks has a length of $\left(w-1\right)2^{L-1}$. A conventional deep convolution layer that raises it exponentially rather than increasing weights of the parameters of the network layer. Finally, hierarchical maps of $DCV^{1},DCV^{2},\ldots \ldots ,DCV^{l}$ were obtained based on the coupling coefficients relation on upstream and downstream layers. SoftMax gives the value of the $b_{io}$ set. Currently, $DCV^{1}=\left[dcv_{1}^{l},dcv_{2}^{l},\ldots ,dcv_{n}^{l}\right]\in R^{n*k^{∼}},l\in \left(1,L\right)$ Here, *l*-th convolutional block output given as $R^{n*k^{∼}},l\in \left(1,L\right)$. Each *k* filter operation output is generated as dcv. $COV_{io}$’s value used as final output features. Now obtain $COV^{1}=\left[COV_{1}^{l},COV_{2}^{l},\ldots ,COV_{n}^{l}\right]\in R^{M*d_{V}}$. The convolution terms size is dv and *M* indicates the amount of final convolution. Now, perform the routing $DCV^{l}$ to $COV^{l}$ for final feature extraction and information generation. The predicting vector ${\tilde{dcv}}_{J|l}$ indicates the raw vector feature transformation that is calculated as the multiplication of $dev_{i}$ with $W_{j}$ in (17).

(17) $${\tilde{dcv}}_{J|l}=dcv_{i}*W_{j}$$

By increasing small vectors and decreasing large vectors into unit vectors, this strategy improves the information exchange efficiency of the complicated routing method. To compute the medium step, we used an iteratively layered routing strategy over multilayered dilated convolution layers.

Here, softmax routing function is $srf_{ij}$ and its modification with dcv is set to $a_{ij}$ agreement. This computation has used Equation (18).

(18) $$a_{ij}=cov_{i}*{\tilde{dcv}}_{J|l}+srf_{ij}$$

Typically, the dilated convolution operation enables more efficient, as well as scalable, convolution routing. Algorithm 3 describes the hierarchical routing scheme of the ADCLR model. In this stage, the autonomous final convolution layer computed as $COV^{1}=\left[COV_{1}^{l},COV_{2}^{l},\ldots ,COV_{n}^{l}\right]\in R^{M*d_{V}}$. In a unified COV, we summed the results of the final convolution in (19).

(19) $$COV^{1}=\left[COV^{l},COV^{l},\ldots ,COV^{l}\right]$$

The action will be passed throughout the hierarchical layer after it is performed. Extracted features $\left[COV^{l},COV^{l},\ldots ,COV^{l}\right]$ of dilated convolution will be assigned.
**Algorithm 3** Feature Extraction Algorithm of DCLR Model*Data: Input synthetic image Tomato disease with calss label**Result : Extract features F_i_ fromTomato leaf disease data**Preprocess Image j to analysis*Process image features$:X=x_{1},x_{2},....,x_{n}\in R^{n*d}$ *here d is dimension*Get dilated convolutional output*Process iteration in dilated CNN***for***each iteration l in range(*0*, l)***do**    **for** *each iteration n in range(*0*, N)* **do**       $DCV^{1}=\left[dcv_{1}^{l},dcv_{2}^{l},....,rv_{n}^{l}\right]\in R^{n*d^{∼}},l\in \left(1,L\right)$       **for** *w in range(*0*,w)* **do**           $dcv_{t}^{l}=ReLU(W^{l}⊕{\left[dcv_{t+1r}^{l-1}\right]}_{t=0}^{w-1})$           $W^{l}\in R^{k*w*k},W^{l}\in R^{k*w*d^{∼}}$           *Here L is number of layers*       **end for**    **end for****end for***Process Dynamic convolution network***for***each iteration i in range(*0*, N)***do**    **for** *each iteration j in range(*0*, N)*
    **do**       $COV_{j}=\sum_{i=1}^{m}cov_{ij}*{\tilde{dcv}}_{J|l}$       ${\tilde{dcv}}_{J|l}=dcv_{i}*W_{j}$       $a_{ij}=COV_{i}*{\tilde{dcv}}_{J|l}+srf_{ij}$    **end for****end for**Execute hierarchical attention mechanism**for** each iteration i in range(0,N) **do**    **for** each iteration j in range(0,N) **do**       **for** each iteration k in range(0,N) **do**          $e_{i}=a\left(q,a_{ij}\right)$          $a_{ij}=\frac{exp(e_{i})}{\sum_{k}exp(e_{k})}$          $ACOV_{i}=q^{T}a_{ij}$       **end for**   **end for****end for***Process features***for***each iteration i in range(*0*, N)***do**   $F_{i}=\sum_{i}ACOV_{i})$**end for**

#### 3.6.2. Hierarchical Attention Layer

This important layer provide a specified and attention aggregation real variable by using each target convolution as input. Algorithm 3 describes the hierarchical attention routing scheme of the ADCLR model. For each target convolution $cov_{i}\in R^{dv}$ in COV, we evaluate attention $a_{ij}$, which produced and will be utilized in the classification layer. Figure 6b presents the attention mechanism. The attention task is computed as given in Equations (20) and (21)

(20) $$\begin{array}{c} e_{i}=a\left(q,a_{ij}\right) \end{array}$$

(21) $$\begin{array}{c} a_{ij}=\frac{exp(e_{i})}{\sum_{k}exp(e_{k})} \end{array}$$

Wherein *q* is a training programme pattern vector, as well as k is the likelihood of convolution pool COV inside the entire pool, the probability of convolution pool COV in the entire pool is presented. After obtaining image features, the weighted total is subsequently applied to the overall target dilated convolution layers in the downstream pattern, resulting in a stationary attention aggregation variable. Figure 6b depicts process of *a* attention mechanism. Finally, the extracted features of the attention-based dilated convolutional are computed as the Equation (22)

(22) $$\begin{array}{c} ACOV_{i}=q^{T}a_{ij} \end{array}$$

According to the the Equation (22), extracted features $ACOV_{i},ACOV_{2},\ldots ,ACOV_{n}$ of the attention mechanism is transformed to $F_{1},F_{2},\ldots ,F_{n}$ for the logistic regression classifier to be classified.

#### 3.6.3. Classification Layer

The main classification procedures presented in the Algorithm 4 after the ADCLR method was trained with a train and validation set of data.

At this point, the ADCLR model uses the LR method to classify images based on the extracted features. LR is a multi-label classification system. The tomato leaf disease target class is predicted at the end of the process. The proposed model’s validation is assessed using various performance evaluation metrics and comparisons on disease images.
**Algorithm 4** Main Classification Algorithm of ADCLR Model Using Logistic Regression*Data: Input F_i_ features of attentive dialted cnn**Result: Prediction Tomato leaf disease**Get extracted features F_1_, F_2_, ...., F_i_ of Dilated CNN**Assign extracted features F_1_, F_2_, ...., F_i_ to x_1_, x_2_, ...., x_i_**Assign target features to y_1_, y_2_, ...., y_i_**Set max iteration as I_max_**Set Augmented Weight Matrix, θ* = 1*Set f unction f or costcalculation L*(*θ*) = 0.*Mapping input f eatures*.*Update a Augmented Weight Matrixθusing*$\theta_{l}\left(n\right)=\theta_{l}\left(n-1\right)=\alpha \times v_{j}$Calculate the Cost function or average costL(θ)using$L\left(\theta \right)=L\left(\theta \right)+\left(-\frac{1}{m}\right)*(\sum_{i=1}^{m}y^{i}*log\left(h_{\theta }*\left(x^{\left(i\right)}\right)\right)+\;(1-y^{\left(i\right)}*log\left(1-h_{\theta }*\left(x^{\left(i\right)}\right)\right)$**if**$\left|l\left(\theta \right)\right|\leq \in \left(or\right)N=N_{max}$ **then**    Find optimum weight of theta**else**    *Update the Augmented Weight Matirxθ***end if***Optimum weights are obtained for θ**Find prediction*$o=sigmoid(\theta^{T}x^{i})$$o=\frac{1}{\left(1+e\theta^{T}x^{i}\right)}$*Execute prediction function with class probability*p(y|I)=p(y|o)

The goal of this layer is to compute the probabilistic model using the formula p(y|S), at which *y* is the class predicted. The vector o is given to the multi-layer classification via the logistic regression function for the fixed-length and care-oriented aggregates. The classification Algorithm 4 operation on tomato leaf disease image data is described in this section. This algorithm receives raw data as input and predicts tomato leaf disease and target classes. The tomato leaf disease image is initially collected and preprocessed. The fully extracted feature from the attentive dilated CNN is transmitted to the attentive hierarchical layer. In the last layer, the logistic regression is used to predict ten types of tomato leaf disease based on the attentive dilated CNN features. Here, Algorithm 4 is the LR classification algorithm that is used for tomato leaf disease classification with the ADCLR model.

First take $F_{i}$ preprocessed features from dilated the CNN layer. Initialize the parameters of the logistic regression classification algorithm. Calculate the $\left(\theta^{T}x\right)$ value of the LR classifier then execute the sigmoid function. Then, obtain output *o* with the computional fuction as the Equations (23) and (25):

(23) $$o=sigmoid(\theta^{T}x)$$

(24) $$o=\frac{1}{\left(1+e\theta^{T}x\right)}$$

Now, execute the prediction function with class probability as (23) follows:

(25) p(y|I)=p(y|o)

**Figure 7:**
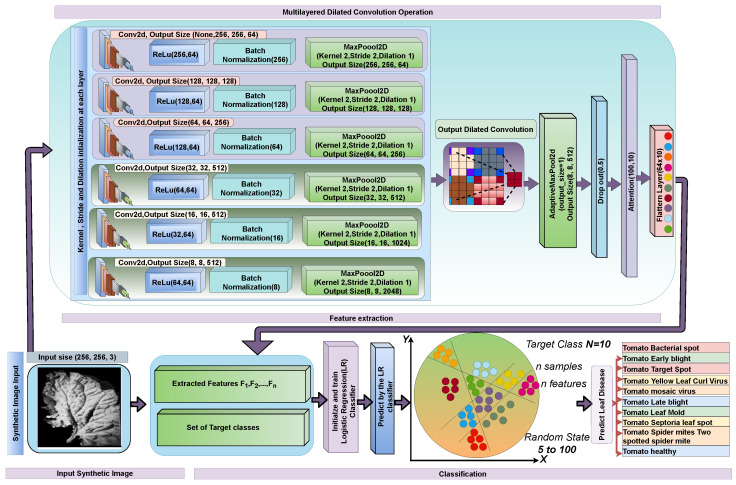
Overall operational process diagram of our ADCLR model.

Figure 7 depicts the operating phase of our concept. From the source term through the prediction or classification algorithm, the mechanics of each layer are depicted in this figure. In one direction, the created output travels through the next processing layer input. To evaluate the performance of the model in the real world, we will use data from tomato leaf disease. The tomato leaf disease image is initially preprocessed as a raw image.

### 3.7. Evaluation Metrics

To evaluate our model performance, we use a performance evaluation matrix named as accuracy, precision, and recall, our used metrics equations and computation are given in Equations (26)–(28).

A. Accuracy
  The average of all true cases is used to determine the Accuracy of the prediction. It is calculated with the specified equation:
  (26) $$\mathrm{Accuracy}=\frac{\left(\mathrm{True}\;\mathrm{Positive}\;+\;\mathrm{True}\;\mathrm{Negative}\right)}{\mathrm{True}\;\mathrm{Positive}\;+\;\mathrm{True}\;\mathrm{Negative}\;+\;\mathrm{Fasle}\;\mathrm{Positive}\;+\;\mathrm{Fasle}\;\mathrm{Negative}}$$
B. Precision
  The amount of true positives divided by the total of positive predictions is known as Precision. The following equation shows the calculation of Precision.
  (27) $$\mathrm{Precision}=\frac{(\mathrm{True}\;\mathrm{Positive})}{\mathrm{True}\;\mathrm{Positive}\;+\;\mathrm{False}\;\mathrm{Positive}}$$
C. Recall
  The Recall is a measurement of how well our model detects True Positives. As a result, Recall informs us how many tomato plants we accurately identified as having leaf disease out of those that have it.
  (28) $$\mathrm{Recall}=\frac{(\mathrm{True}\;\mathrm{Positive})}{\mathrm{True}\;\mathrm{Positive}\;+\;\mathrm{False}\;\mathrm{Negative}}$$
D. F1 Score
  The F1 score elegantly summarizes a model’s predictive efficiency and measured by two normally competing metrics, precision and recall.
  (29) $$\begin{array}{c} F1=2*\frac{\left(\mathrm{Precession}*\mathrm{Recall}\right)}{\mathrm{Precession}\;+\;\mathrm{Recall}} \end{array}$$

#### Experimental Setup

After the successful preprocessing procedures (BF filtering and Otsu, Segmentation method), we generated the synthetic image using CGAN. Then, we fed this to the proposed methodology developed with attention-based dilated CNN with logistic regression. We fine-tuned the proposed model in our trials to demonstrate the performance of our model. To evaluate the model, we used a binary cross-validation strategy. Indeed, we divided the dataset into 80% training and 20% validation, with 1000 images used for testing. The remaining 20% was utilized to validate the model before it was evaluated. The attention-based dilated CNN feature extraction model was trained with 100 epochs and 32 mini-batches. To reduce the loss (L), the Adam optimizer was used with only a $1\times 10^{-3}$ learning rate. We employed an L2 regularization and a dropout technique with a probability of dropping of 0.5 to offset the effect of the overfitting problem during training. The number of layers of the dilated CNN that was gradually configured to extract features from tomato leaf disease images. The model was implemented using the Python programming language as well as the Google colab framework. The categorical cross-validation was performed on a computer with five CPUs (Intel(R) 3.60 GHz), 32 GB of RAM, and Windows 8 to 10. Figure 7 and Table 2 clearly describes the model’s internal structure.

## 4. Result Analysis

This section explains the experimental analysis in detail, including qualitative and comparative analysis. The training set performs somewhat better than the validation set, as well as the model accumulating to a steady value, showing that the parameters used to train the model really are not excessive. In the validation model, the suggested technique achieves stable classification performance with good accuracy. In Table 3, Table 4 and Table 5 we use alphabet A to J as A: Bacterial Spot, B: Early Blight, C: Target Spot, D: Yellow Leaf Curl Virus, E: Mosaic Virus, F: Late Blight, G: Leaf Mold, H: Septoria Leaf Spot, I: Two spotted Spider Mite and J: Healthy.

### 4.1. Qualitative Analysis

In this qualitative analysis, a comprehensive experimental analysis is shown. Here, Table 3 shows the training performance of the proposed ADCLR model on ten disease classes of the tomato leaf dataset. The Table 3 demonstrated that our model performs the same on train and validation data.

Table 4 shows the validation performance of the proposed ADCLR model on ten disease classes of tomato leaf.

Table 5 shows the testing performance of the proposed ADCLR model on ten disease classes of tomato leaf. It is clearly shown that the testing accuracy of the proposed model is slightly lower than the train and validation performance, because we use totally different image for testing the model.

**Figure 8:**
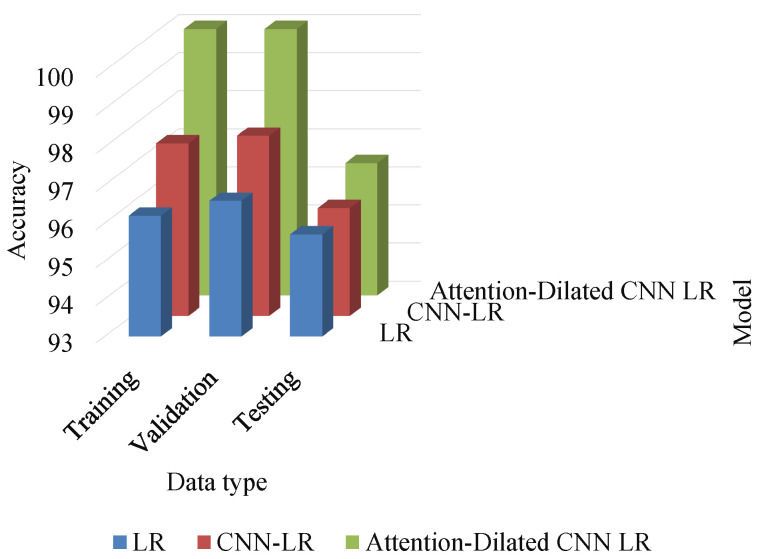
The training, validation, and testing performance of ADCLR model.

The experimental analysis demonstrated that the testing performance is slightly lower than the training and validation performance. Our ADCLR model achieved 100%, 100%, and 96.60% accuracy on train, validation, and test data, respectively. We also implemented some popular conventional methods and used the same parameter tuning in each model. Based on the results shown in the Table 6, it is clearly shown that our attention-based dilated CNN with logistic regression (ADCLR) model outperforms the other method we implemented in this study for tomato leaf disease detection. The comparative result of LR, CNN-LR and Attention based Dilated CNN-LR method is showed in the bar chart in Figure 8. This figure clearly present that our ADCLR method got higher accuracy that compared method.

### 4.2. Confusion Matrix

**Figure 9:**
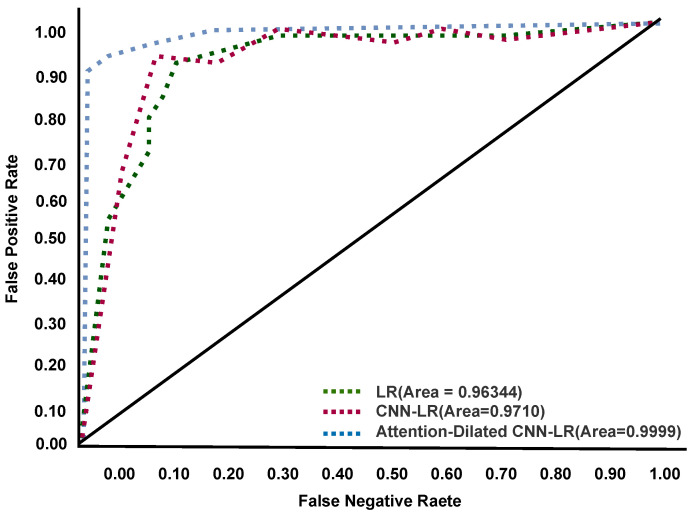
ROC curve during training.

**Figure 10:**
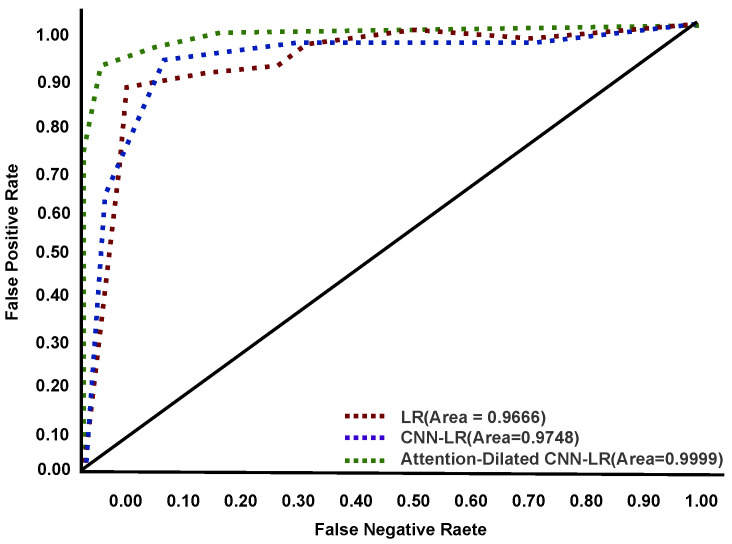
ROC curve during validation.

**Figure 11:**
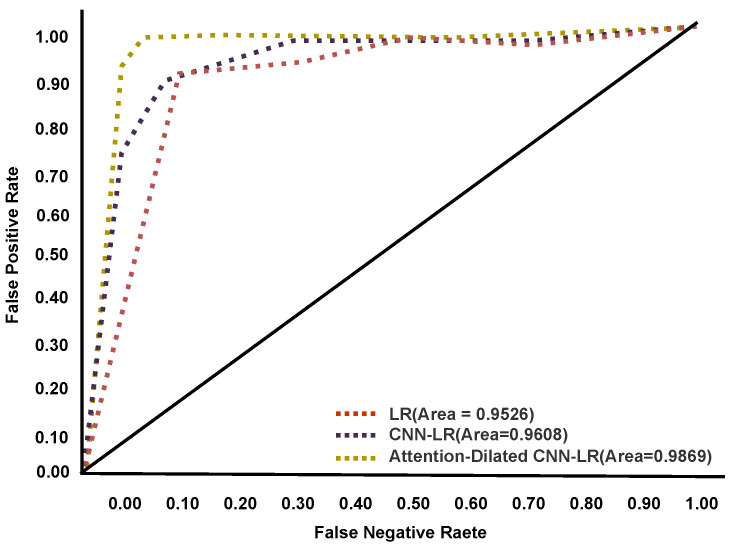
ROC curve during testing.

The Area Under the Curve (AUC)—receiver operating characteristic curve (ROC) curve is a performance efficiency measurement technique for multiclass classification. AUC indicates the degree or measurement of separability, whereas ROC is a probability curve. It indicates how well the model can distinguish among categories based on the training, validation, and testing data performance. The ADCLR model generates the following graphical ROC result on tomato leaf disease data. The ROC curve performances are visualized in Figure 9, Figure 10 and Figure 11 during training, validation, and testing. In these figures, the *x*-axis indicates a false-positive rate and the *y*-axis indicates a false-negative rate. Our ADCLR model shows better ROC performance over other models. The ADCLR model obtained 0.999 area value of ROC for both of training and validation, and 0.9869 on testing data.

**Figure 12:**
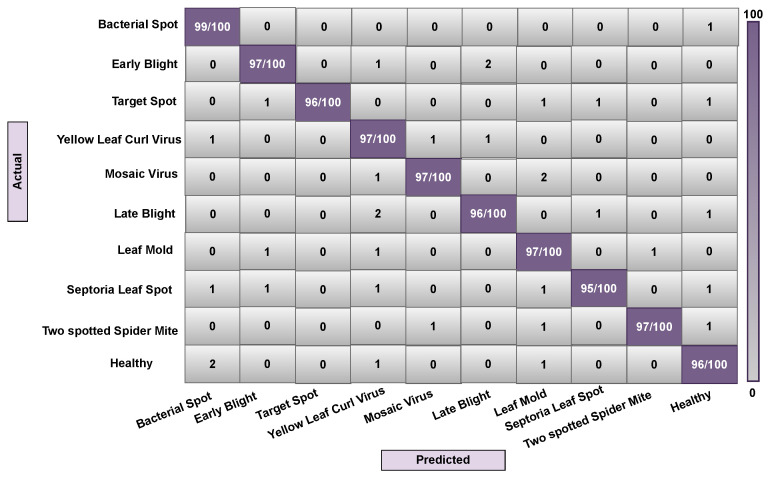
Confusion matrix of ADCLR method during testing.

**Figure 13:**
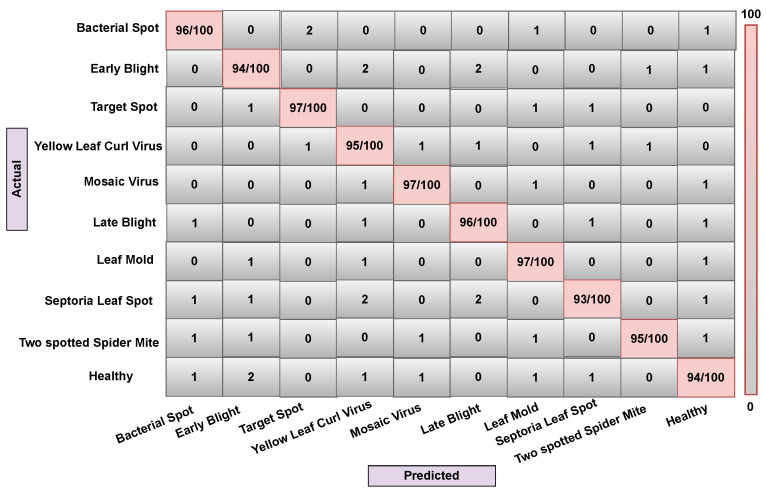
Confusion matrix of CNN-LR method during testing.

**Figure 14:**
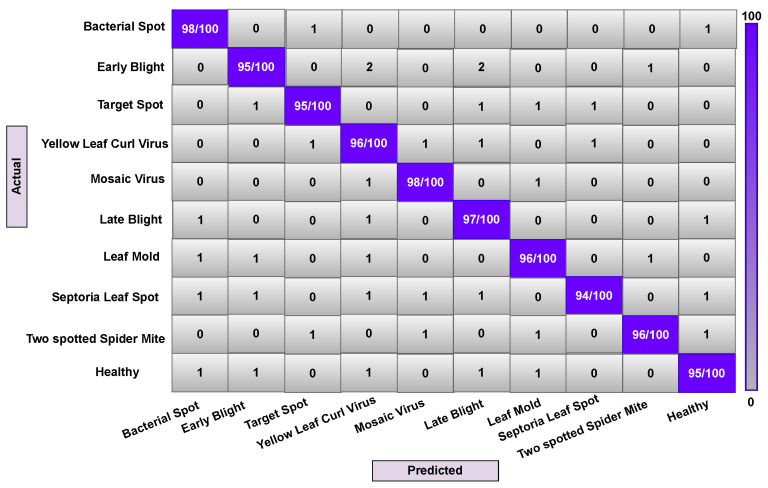
Confusion matrix of LR method during testing.

In some contexts, the classifier can become confused when dealing with many classes with comparable features. A confusion matrix could be used to visually measure a model’s classification performance. This experiment was conducted using ten different types of tomato leaf disease. Since the leaf disease image samples are created from the leaf area and are very unstable, the low resolution and poor area selection of the images can result in noisy leaf images, causing the classifier to become confused in some circumstances. We implemented our model (Attention-Dilated CNN LR) and related models (LR and CNN-LR) on train, validation, and test data to generate a confusion matrix. Here, Figure 12, Figure 13 and Figure 14 are generated based on LR, CNN-LR, and Our (Attention dilated CNN-LR) model. All right predictions seem to be on the diagonal, while all wrong predictions are off the diagonal.

An overall description of the data analysis during training is shown in Figure 12. The LR algorithm correctly predicts 952 observations out of 1000 observations, the CNN-LR algorithm correctly predicts 960 observations out of 1000 observations. The proposed attention-based dilated CNN-LR algorithm correctly predicts 966 observations out of the same number of observations. Our proposed ADCLR algorithm performs better in prediction on validation train data as shown in Figure 12, Figure 13 and Figure 14. So, the confusion matrix also indicates that the proposed Attention-Dilated CNN-LR architecture is more accurate than the LR and CNN-LR model. It also helps to overcome the limitations of LR algorithms and works better than CNN-based feature extraction for tomato leaf disease detection.

### 4.3. Comparisons with State-of-the-Art Methods

**Figure 15:**
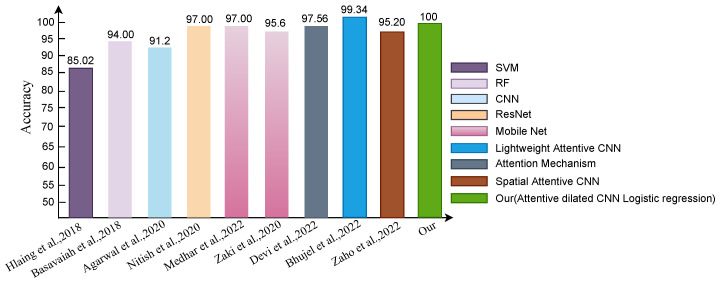
Comparison of state of the art method [28,29,34,37,39,48,50].

This section will discuss all current methodologies, as well as the performance of our proposed method, in classifying the tomato leaf disease Plant Village database. Table 7 categorizes all of the compared methods into three groups: traditional machine learning (ML), deep learning (DL), as well as Deep learning + Machine learning (DLML). Table 7 presents different existing methods performance with their features, data, model, and evaluation metric result. From the comparative table, it is shown that SVM with SIFT features had 85% accuracy [29], RF [29] with Hue and histogram color features having 94% accuracy, the ResNet model obtained 97% accuracy [39]. On the other hand, Machine learning with a deep learning classifier achieved sightly higher accuracy than conventional the ML or DL method. MobileNet and NasNet feature extractor with Logistic Regression got 97% accuracy [28]. Fine-tuned MobileNetv2 obtained 95.6% accuracy. The SVM algorithm got very low accuracy of 85.02% but this method is fast [29]. A maximum of 94% classification accuracy is obtained with the random forest method [30]. Another attention-based method proposed by Devi et al. [48] that used the Salp Swarm Algorithm had 97.56% accuracy to predict five types of tomato leaf disease. The Lightweight Attention-Based CNN mechanism [49] to classify ten types of tomato leaf disease. This method obtained 99.34% accuracy but it has slightly higher time complexity than conventional methods such as CNN [37] and SVM [29]. In 2022, Zhao et al. [50] developed a method utilizing Spatial attention with CNN that had 95.20% accuracy but this has a weakness in generability. We have also shown the comparative performance of the state-of-the-art method in Figure 15.

#### Comparison of Pre-Network Recognition Accuracy

**Figure 16:**
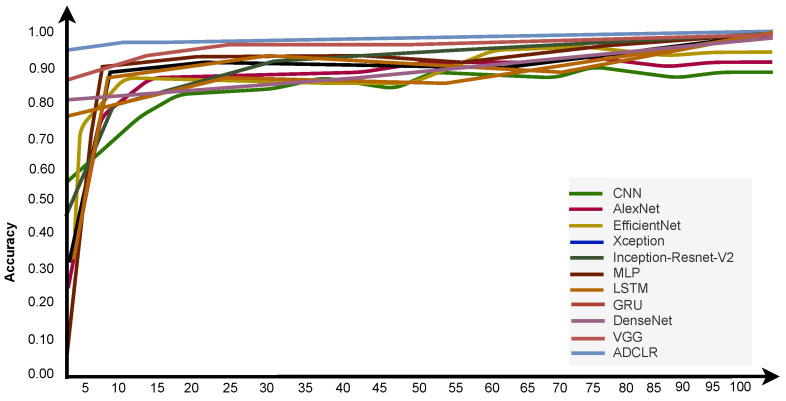
Accuracy comparison of the implemented deep learning models during training.

We also implemented some conventional methods with the same parameter tuning and input size to check the validation of our model. Based on this table, it is clearly shown that our ADCLR method performs better than other implemented methods. We also calculated the executing time of the widely used popular deep learning model for feature extraction. We run and check the time of execution on train, validation, and test sample data. Figure 16 shows the accuracy of different deep learning models during training. In the figure, the *x*-axis indicates the number of epochs, and the y-axis indicates the accuracy. The graphical line shows the performance of compared models and the proposed model. Figure 16 clearly indicates that our model performs better than the conventional method.

An overall performance (accuracy) comparison of the ADCLR model with most common and related models is clearly shown in Figure 16. Our method got higher accuracy than other implemented methods with the same parameter tuning. Additionally, Table 8 is generated based on our manual implementation with the same parameter tuning and shows that our ADCLR model performance is better compared to the conventional approach.

**Figure 17:**
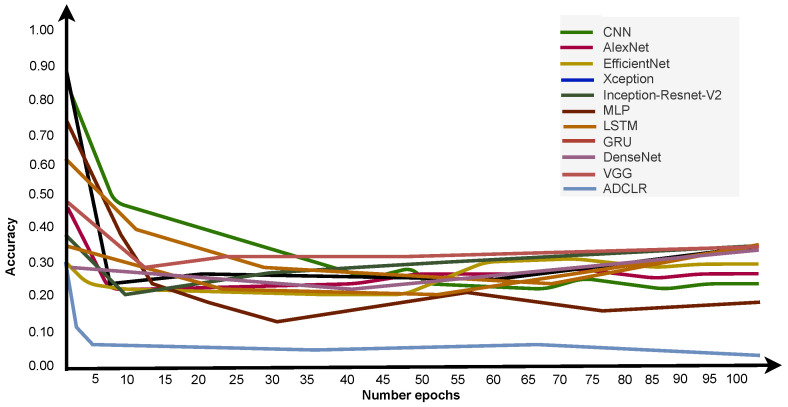
Loss comparison of the implemented deep learning models during training.

Figure 17 shows the loss of different deep learning models during training. In the figure, the *x*-axis indicates the number of epochs, and the *y*-axis indicates the loss. The graphical line shows the performance of compared models and the proposed model. Figure 17 clearly indicates that our model’s loss is less than the conventional method. The categorical cross entropy loss of our ADCLR method is 0.07.

### 4.4. Discussion

The proposed method performed image preprocessing using bilateral filtering (BF), segmentation using Otsu’s thresholding, synthetic image generation, feature extraction using attention-based dilated CNN, and classification using logistic regression. The hyper-parameter tuning on logistic regression (LR) seeks the fine change of the hyper-parameters of the attention-based dilated CNN model of feature extraction in such a way that the classification performance is improved to the highest extent possible. To ensure that the attention-based dilated CNN-LR model performs effectively, a complete simulation analysis is performed. The experimental results suggest that the ADCLR model outperforms contemporary state-of-the-art methods on a variety of measures as shown in Table 8. In the future, advanced DL-based image segmentation techniques will be used to improve the detection efficiency of the ADCLR method.

The techniques of our successive preprocessing are computed from the original normal Tomato leaf disease images. The preprocessing helps to extract more precise features from the images. Then, we use the CGAN model to generate the synthetic image to handle imbalance and noisy or wrongly labeled data to obtain good prediction results. The synthetic image is used in the attention-based dilated CNN layer for feature extraction. This aid of this technique is to reduce the misclassification issues and improve performance. In our method, the Bilateral filtering technique helps to remove the noise of the tomato leaf disease image. As a result, Otsu’s method of image segmentation is useful for handling the noise of tomato leaf disease images. Otsu’s image segmentation technique is faster and simpler than other methods [56].

In this proposed ADCLR method for feature extraction, which leverages the attention-based hybrid dilated CNN approach. By dynamically converting its hierarchical system into a deep convolution, we present a new hybrid model for optimizing learning structure, extracting features, classification and analyzing tomato leaf disease. It can automatically extract the hierarchical representations of tomato leaf disease features in order to fully leverage the features. Our hybrid neural network convolution model successfully obtains implicit and relevant feature information. The dilated convolution network can extract informative information about the features. Our hybrid approach, which uses dilated CNN and is based on a paradigm that includes hierarchical self-dilation approaches, provides a reduction in training time and a clear network structure to boost performance. The efficacy of the convolution network dynamic routing algorithm has been increased and an improved convolutional network dynamic convolution method improves the efficacy of efficient routing tuning convolution.

In our ADCLR model, the attention layer simply concentrates on the memory block instead of focusing on the entire feature space. The attention mechanism has the advantage of dramatically reducing the number of parameters and sharing the weights among diverse regional places.

The limitations of previous classification systems are well outlined in the literature section, and our novel ADCLR model is aimed to overcome some of the weaknesses of the compared method. The proposed ADCLR model is evaluated and compared to a number of existing model benchmarks. The proposed ADCLR model achieved an accuracy of 100% in training, 100% in validation, and 96.6% in testing on the PlantVillage tomato leaf disease dataset. This method predicts ten categorizations of the tomato leaf dataset. The experimental analysis of this study showed that our method outperforms over a number of competing baselines and produces a number of cutting-edge outcomes.

### 4.5. Real Time Test Result on New Image

**Figure 18:**
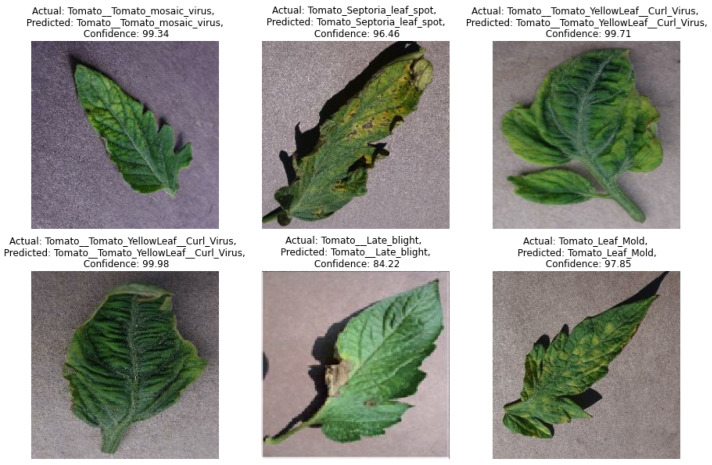
Test Result in Leaf disease detection by our DCLR method.

To study the robustness of the proposed model in a real-time application, we used 1000 non-trained images. In Figure 18, we show the predicted class confidence of the new image of tomato leaf disease. In this figure, we show the result of our model on six images. In this figure, it is clearly shown that the prediction confidence of our model is almost 0.99 for all the new and non-trained tomato disease image samples.

### 4.6. Complexity Analysis

**Figure 19:**
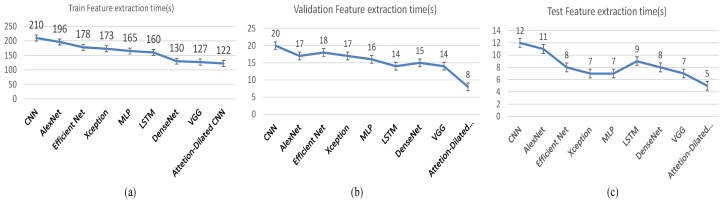
Comparative feature extraction time during (**a**) Training, (**b**) Validation, (**c**) Testing.

To ensure the superiority of the proposed strategy in terms of execution time, we constructed a hybrid deep learning ADCLR model that utilized attention-based dilated CNN to extract informative features. During the testing set, the model’s recorded running speed is higher than any other implemented deep learning approach. This method does not use transfer learning or a convolutional neural network (CNN) in feature extraction because its training time is long (see Figure 19). However, we apply a dilated convolution operation with a multi dilation mechanism with attention that uses only the convolutions required for deep feature extraction that helps to reduce the overall computational complexity. The training, validation set has a 122.8 and 5 s run time, respectively (see Figure 19). Figure 19 shows that our attention-based dilated CNN takes less time (122 s) in training whereas CNN takes 210 s. Similarly, our model’s attention-based dilated CNN takes less time of 8 and 5 s for feature extraction of validation and testing data, respectively. In normal convolution layers, convolution kernels are also interconnected and all are convoluted. Furthermore, the addition of attention-based dilated portions, minimizes the processing complexity of the convolutions. The added attention mechanism uses less parameters to select most important and relevant features from the selected region. The time complexity of our model is lower than compared methods.

### 4.7. Limitation and Future Work

Despite the outstanding performance of the proposed ADCLR model for tomato leaf disease detection, there are a few flaws in the research as well. Firstly, this study only focuses on ten types of tomato leaf disease images for classification, other category of tomato plant leaf disease images did not analyzed. Secondly, Our proposed model validity has been investigated on only the PlantVillage tomato leaf dataset. In a further study, we intend to provide larger and more diverse datasets to test the proposed model and continuously improve the network system’s generalization capacity. We will also work to refine our model so that it may be applied to other datasets including tomato or other leaf disease. Additionally, one of the primary drawbacks of the suggested detection method is that the image used in our experiment was taken in a lab setting. However, our method might be improved to support an integrative plant disease detection system that works in real-world scenarios. However, further work is needed to make this model more advanced to classify broadening categories of plant diseases and automatically recognizing the many stages of the disease, as well as complementing images of leaf diseases in real surroundings.

**Figure 20:**
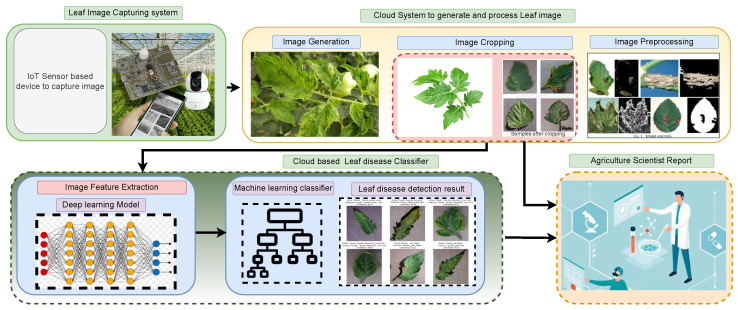
Cloud based Leaf disease detection.

Figure 20 shows a possible cloud-based tomato leaf disease detection system that can be utilized on mobile phones. The cloud-based system could be able to collect and process the image of tomato leaf disease from the real-time field. The processed data will be interpreted by the cloud-based DL system, and the results are delivered to the agriculture scientist with minimal human effort. Finally, the obtained results will be sent to the farmer’s mobile phone after being verified by the practitioner. The processing costs of the DL model, as well as the data dimension, impact the system’s feasibility. We have a plan to reduce the time complexity and space complexity in future development with more adaptability and generability.

## 5. Conclusions

The early tomato leaf disease diagnosis method has a great effect on the quality and quantity of tomato production. Traditional methods for detecting tomato disease are time-consuming, labor-intensive, and subjective cost. This study has designed a hybrid architecture (ADCLR) based on attention and dilated convolutional layers with an LR classifier.At first tomato leaf disease images are preprocessed (using nilateral fileterng and otsu segmentation) properly and then we used our Conditional Generative Adversarial Network(CGAN) to generete synthetics tomato leaf disease image. The informative and relevant features from the images were extracted quickly using the attention-based dilated convolutional layers. Then LR has been used to classify the extracted feature. Ten types of tomato leaf disease have been analyzed in this study. Three related classifiers have also been implemented (LR, CNN-LR, and Attention-Dilated CNN-LR) in this study. The validation of the method has been tested using 1000 non-trained images sample. In comparison with other state-of-art methods (CNN, AlexNet, Efficient Net, Xception, MLP, LSTM, GRU, DenseNet, and VGG), our proposed method has achieved higher performance for the tomato leaf disease detection. Our method will work for more types of diseases of plant leaves in the future. We have a plan to reduce the time complexity and space complexity in future development. In a further study, we intend to design a cloud-based artificial intelligence (AI) system using deep learning techniques with more data variants. 

## Figures and Tables

**Table 1:** The types of Tomato leaf disease.

Leaf Disease Class	Amount	Percentage %
Bacterial spot	2126	13.29
Early blight	1000	6.25
Target Spot	1403	8.77
Yellow Leaf Curl Virus	3107	19.43
Mosaic virus	372	2.32
Late blight	2005	12.54
Leaf Mold	951	5.94
Septoria leaf spot	1760	11
Two spotted spider mite	1675	10.48
Healthy	1590	9.94
Total	15,989	100

**Table 2:** Network parameter of the proposed model.

Layer No	Layer Name	Layer Information	Image Size	Output Size
01	2d Convolution	Dilated Convolution	256 × 256 × 3	(None, 256, 256, 64)
02	Activation	ReLU	256 × 253 × 3	(256, 64)
03	Normalization	Batch Normalization (256)	256 × 253 × 3	(256, 256, 64)
04	Pooling	MaxPoool2D (Kernel 2, Stride 2, Dilation 1)	256 × 253 × 3	(256, 256, 64)
05	2d Convolution	Dilated Convolution	256 × 253 × 3	(128, 128, 64)
06	Activation	ReLU	256 × 253 × 3	(128, 64)
07	Normalization	Batch Normalization (128)	256 × 253 × 3	(128, 128, 64)
08	Pooling	MaxPoool2D (Kernel 2, Stride 2, Dilation 1)	256 × 253 × 3	(128, 128, 128)
09	2d Convolution	Dilated Convolution	256 × 253 × 3	(64, 64, 64)
10	Activation	ReLU	256 × 253 × 3	(64, 64)
11	Normalization	Batch Normalization (64)	256 × 253 × 3	(64, 64, 64)
12	Pooling	MaxPoool2D (Kernel 2, Stride 2, Dilation 1)	256 × 253 × 3	(64, 64, 256)
13	2d Convolution	Dilated Convolution	256 × 253 × 3	(32, 32, 64)
14	Activation	ReLU	256 × 253 × 3	(32, 64)
15	Normalization	Batch Normalization (32)	256 × 253 × 3	(32, 32, 64)
16	Pooling	MaxPoool2D (Kernel 2, Stride 2, Dilation 1)	256 × 253 × 3	(32, 32, 512)
17	2d Convolution	Dilated Convolution	256 × 253 × 3	(16, 16, 64)
18	Activation	ReLU	256 × 253 × 3	(16, 64)
19	Normalization	Batch Normalization (16)	256 × 253 × 3	(16, 16, 64)
20	Pooling	MaxPoool2D (Kernel 2, Stride 2, Dilation 1)	256 × 253 × 3	(16, 16, 1024)
21	2d Convolution	Dilated Convolution	256 × 253 × 3	(8, 8, 64)
22	Activation	ReLU	256 × 253 × 3	(8, 64)
23	Normalization	Batch Normalization (16)	256 × 253 × 3	(8,8, 64)
24	Pooling	MaxPoool2D (Kernel 2, Stride 2, Dilation 1)	256 × 253 × 3	(8, 8, 2048)
25	Activation	ReLU	256 × 253 × 3	(256, 64)
26	Pooling	AdaptiveMaxPool2d	256 × 253 × 3	(8, 512)
27	Dropout	Drop out (0.5)	256 × 253 × 3	
28	Attention	Attention	256 × 253 × 3	(100,10)
29	Flattern	Flatten Layer	256 × 253 × 3	(64 × 10)
30	Logistic Regression	Logistic Regression (LR) Classifier (N = 10, Number of features, Random State = 100)	256 × 253 × 3	10

**Table 3:** Training performance of ADCLR model.

Model LR	Metrics	Training Performance									
		A	B	C	D	E	F	G	H	I	J
LR	Accuracy	0.96	0.95	0.96	0.97	0.98	0.96	0.97	0.95	0.97	0.96
	Precession	0.98	0.95	0.94	0.98	1.00	0.96	0.98	0.93	0.96	0.99
	Recall	0.96	0.95	0.96	0.97	0.98	0.96	0.92	0.98	0.98	0.98
	F1	0.96	0.98	0.93	0.96	0.99	0.96	0.95	0.96	0.97	0.98
CNN-LR	Accuracy	0.97	0.97	0.96	0.97	0.97	0.97	0.98	0.98	0.97	0.96
	Precession	0.99	0.95	0.94	0.98	1.00	0.96	0.98	0.93	0.96	0.99
	Recall	0.99	0.93	0.98	1.00	0.97	0.96	0.94	0.98	0.99	0.98
	F1	0.98	0.95	0.96	0.99	0.98	0.96	0.94	0.96	0.97	0.98
Attention-Dilated CNN- LR	Accuracy	1.00	1.00	1.00	1.00	1.00	1.00	1.00	1.00	1.00	1.00
	Precession	1.00	1.00	1.00	1.00	1.00	1.00	1.00	1.00	1.00	1.00
	Recall	1.00	1.00	1.00	1.00	1.00	1.00	1.00	1.00	1.00	1.00
	F1	1.00	1.00	1.00	1.00	1.00	1.00	1.00	1.00	1.00	1.00
Training supports as 80% data		1701	800	1123	2486	298	1604	761	1416	1340	1272

**Table 4:** Validation performance of ADCLR model.

Model	Metrics	Validation Performance									
		A	B	C	D	E	F	G	H	I	J
LR	Accuracy	0.97	0.96	0.97	0.97	0.98	0.96	0.97	0.95	0.97	0.96
	Precession	0.99	0.96	0.95	0.99	1.00	0.96	0.98	0.94	0.96	0.99
	Recall	0.97	0.98	0.96	0.97	0.99	0.96	0.92	0.98	0.98	0.98
	F1	0.96	0.98	0.94	0.98	0.99	0.97	0.98	0.97	0.97	0.98
CNN-LR	Accuracy	0.98	0.97	0.97	0.98	0.98	0.98	0.98	0.97	0.97	0.96
	Precession	0.99	0.96	0.98	0.99	1.00	0.97	0.97	0.97	0.98	0.99
	Recall	0.99	0.94	0.99	1.00	0.97	0.99	0.98	0.98	0.99	0.98
	F1	0.98	0.95	0.96	0.99	0.99	0.98	0.99	0.99	0.98	0.98
Attention-Dilated CNN-LR	Accuracy	1.00	1.00	1.00	1.00	1.00	1.00	1.00	1.00	1.00	1.00
	Precession	1.00	1.00	1.00	1.00	1.00	1.00	1.00	1.00	1.00	1.00
	Recall	1.00	1.00	1.00	1.00	1.00	1.00	1.00	1.00	1.00	1.00
	F1	1.00	1.00	1.00	1.00	1.00	1.00	1.00	1.00	1.00	1.00
Validation supports as 20% data		425	200	280	621	74	401	190	354	335	318

**Table 5:** Testing performance of ADCLR model.

Model	Metrics	Testing Performance									
		A	B	C	D	E	F	G	H	I	J
LR	Accuracy	0.96	0.94	0.94	0.95	0.97	0.96	0.97	0.93	0.95	0.94
	Precession	0.98	0.91	0.9	0.96	0.98	0.96	0.98	0.95	0.96	0.97
	Recall	0.97	0.89	0.94	0.98	0.98	0.93	0.9	0.92	0.94	0.96
	F1	0.95	0.92	0.95	0.98	0.95	0.94	0.95	0.99	0.96	0.94
CNN- LR	Accuracy	0.98	0.95	0.95	0.96	0.98	0.97	0.96	0.94	0.96	0.95
	Precession	0.99	0.94	0.93	0.97	0.99	0.95	0.97	0.94	0.95	0.98
	Recall	0.97	0.92	0.95	0.99	0.99	0.94	0.91	0.94	0.96	0.97
	F1	0.96	0.93	0.95	0.97	0.97	0.95	0.94	0.98	0.98	0.95
Attention-Dilated CNN- LR	Accuracy	0.99	0.97	0.96	0.97	0.97	0.96	0.97	0.95	0.97	0.96
	Precession	0.98	0.95	0.94	0.98	1.00	0.96	0.98	0.93	0.96	0.99
	Recall	0.98	0.93	0.97	1.00	0.97	0.96	0.92	0.98	0.98	0.98
	F1	0.99	0.95	0.96	0.99	0.98	0.96	0.95	0.96	0.97	0.98
Supports 100 new image per class		100	100	100	100	100	100	100	100	100	100

**Table 6:** The performance comparison of three related models (training, validation and testing).

**Training Performance**			
**Model**	**Input Size**	**Sample**	**Accuracy**
LR	256 × 256 × 3	12,801	96.30%
CNN-LR	256 × 256 × 3	12,801	97.00%
Attention-Dilatd CNN LR(ADCLR)	256 × 253 × 3	12,801	100.0%
**Validation Performance**			
**Model**	**Input Size**	**Sample**	**Accuracy**
LR	256 × 256 × 3	3198	96.6%
CNN-LR	256 × 253 × 3	3198	97.4%
Attention-Dilatd CNN LR(ADCLR)	256 × 253 × 3	3198	100.00%
**Testing Performance**			
**Model**	**Input Size**	**Sample**	**Accuracy**
LR	256 × 256 × 3	1000	95.20%
CNN-LR	256 × 253 × 3	1000	96.00%
Attention-Dilatd CNN LR(ADCLR)	256 × 256 × 3	1000	96.60%

**Table 7:** The performance comparison of the related studies with the proposed approach. DL: Deep Learning, ML = Machine Learning, DLML = Deep learning with Machine Learning.

Type	Author	Method	Features	Class	Samples	Data	Performance
ML	Hlaing et al. [29]	SVM	SIFT and color conversion Features	7	3535	Plant Village (Tomato)	Accuracy 85.02%
	Basavaiah et al. [29]	Random forest and decision tree	Hu Moments, pattern and colour histograms.	5	300	Plant Village (Tomato)	Accuracy 94%(RF) Accuracy 90%(DT)
DL	Agarwal et al. [37]	CNN	CNN model	10	10,100	Plant Village (Tomato)	Accuracy 91.2%
	Nitish et al. [39]	ResNet	ResNet-50 model	6	12,206	Plant Village (Tomato)	Accuracy 97%
MLDL	Medhar et al. [28]	MobileNetV2 or NASNetMobile and Logistic Regression	MobileNetV2 or NASNetMobile feature extractor	6	1,152	Plant Village (Tomato)	Accuracy 97% (MobileNetV2) Accuracy 97% (NASNetMobile)
	Zaki et al. [34]	MobileNetV2	Fine-tune MobileNetV2	4	3471	Plant Village (Tomato)	Accuracy 95.6%
	Devi et al. [48]	Attention mechanism	Dense net with Attention	5	9281	Plant Village (Tomato)	Accuracy 97.56%
	Bhujel et al. [49]	Lightweight Attention-Based CNN	Attentive CNN	10	19,510	Plant Village (Tomato)	Accuracy 99.34%
	Zaho et al. [50]	Spatial attention with CNN	Fully connected layer	10	18,160	Plant Village (Tomato)	Accuracy 95.20%
	Proposed ADCLR (Our)	Attention-Dilated CNN and Logistic Regression with synthetic image	Attention-based Dilated CNN	10	15,989	Plant Village (Tomato)	Accuracy 100.00% F1 100.00% Precession 100.00% Recall 100.00%

**Table 8:** Performance comparison of the implemented conventional models with the proposed ADCLR model.

Model	Input Size	Accuracy	Precessoin	Recall	F1-Score
CNN	256, 256, 3	88.70%	86.76%	88.71%	87.30%
AlexNet	256, 256, 3	91.87%	89.93%	91.88%	90.47%
EfficientNet	256, 256, 3	92.25%	90.31%	92.26%	90.75%
Xception	256, 256, 3	97.61%	95.67%	88.70%	96.21%
Inception-Resnet-V2	256, 256, 3	97.80%	95.86%	95.87%	96.41%
MLP	256, 256, 3	97.99%	96.05%	97.99%	96.59%
LSTM	256, 256, 3	98.50%	96.56%	98.50%	97.11%
GRU	256, 256, 3	98.74%	96.80%	98.75%	97.34%
DenseNet	256, 256, 3	98.88%	96.94%	98.89%	97.48%
VGG	256, 256, 3	99.00%	97.06%	99.01%	97.61%
Dilated CNN-RNN	256, 256, 3	99.15%	97.21%	99.15%	97.75%
ADCLR (Our)	256, 256, 3	100.00%	100.00%	100.00%	100.00%

## Data Availability

We have no availability of data at this time, in future we will provide code and data publicly.
